# Impact of COVID-19 on pediatric dental care in two epicenters: Italy and Brazil

**DOI:** 10.1590/1807-3107bor-2024.vol38.0068

**Published:** 2024-08-05

**Authors:** Camilla Karoline de Carvalho BECKMAN, Valentina LUPPIERI, Letícia Martins PEREIRA, Camila Ribeiro SILVA, Paula Midori CASTELO, Milena CADENARO, Regina Maria Puppin RONTANI, Aline Rogéria Freire de CASTILHO

**Affiliations:** (a) Universidade Estadual de Campinas – Unicamp, Faculdade de Odontologia de Piracicaba, Departamento de Ciências da Saúde e Odontologia Infantil, Piracicaba, SP, Brazil.; (b) Institute for Maternal and Child Health, IRCCS “Burlo Garofolo”, Trieste, Italy.; (c) Universidade Estadual de Campinas – Unicamp, Faculdade de Odontologia de Piracicaba, Departamento de Odontologia Restauradora, Piracicaba, SP, Brazil.; (d) Universidade Federal de São Paulo, Instituto de Ciências Ambientais, Químicas e Farmacêuticas, Departamento de Ciências Farmacêuticas, Diadema, SP, Brazil.; (e) University of Trieste, Department of Medicine, Surgery and Health Sciences, Trieste, Italy.; (f) Indiana University, School of Dentistry, Department of Pediatric Dentistry, Bloomington, IU, USA.

**Keywords:** COVID-19, Pandemics, Pediatric Dentistry, Containment of Biohazards, Infection Control

## Abstract

The study aimed to compare the adherence of Brazilian and Italian pediatric dentists to the biosafety measures and operative protocols recommended by the health authorities during COVID-19 pandemic and to classify the participants according to their risk of infection. An online questionnaire with 34 questions about sociodemographic and occupational data, dental practice organization, biological risk management, and clinical operative protocols was sent to Brazilian and Italian pediatric dentists using a convenience sampling strategy. Chi-square test and multivariate analysis (two-step cluster) were performed (α = 5%). Of 641 respondents (377 Brazilians and 264 Italians), most were female (94% and 70%, respectively), aged 20-39 years (63%), with over 10 years of professional experience (58% and 49%, respectively). Based on adherence to recommended biosafety measures, participants were classified as “safer” (n = 219) or “less safe” (n = 422). Adherence to recommended protocols by the majority of participants resulted in low contagion rates (Brazilians = 5%; Italians = 12.5%). Participants with extensive professional experience in the dental setting exhibited a greater tendency to implement multiple adaptations (three or more) in their practice. Most participants (Brazilians = 92%; Italians = 80.7%) adopted the recommended minimal intervention dentistry approaches, with the use of fissure sealants and the use of non-rotary instruments for caries removal the most frequently techniques used among Brazilians (36%) and Italians (66%), respectively. Two different profiles of pediatric dentists were identified based on the biosafety protocols adopted during the pandemic. In addition, changes were implemented in the dental care provided to children, with focus on the minimal intervention dentistry.

## Introduction

The sudden emergence of the new coronavirus named Severe Acute Respiratory Syndrome Coronavirus-2 (SARS-CoV-2) by the International Committee on Taxonomy of Viruses (ICTV)^1^ at end of 2019 raised significant public health concerns worldwide and brought new doubts and challenges for healthcare professionals.^2,3^ SARS-CoV-2 is responsible for the Severe Acute Respiratory Syndrome (SARS), referred to by the World Health Organization (WHO) as Coronavirus Disease-19 (COVID-19), which causes a wide range of respiratory symptoms and could also be associated with vasculopathy and other complications.^4,5^ Genomic alterations of SARS-CoV-2 have demonstrated that the virus has several highly transmissible variants that increase the risk of its worldwide spread.^6,7^

As COVID-19 is a respiratory disease that is airborne^8-10^, dental professionals are at the top of the list of the healthcare professionals at higher risk of contagion, as reported by the New York Times in 2020.^11^ The frequent use of rotatory handpieces that produce potentially infectious aerosol^12^ and face-to-face working conditions increase the risk of exposure to the virus for both patients and dental professionals.^8^ Particular attention must be paid to pediatric dentistry, since in children, the COVID-19 infection is mostly asymptomatic or has mild or moderate symptoms that are not always easily detectable. Because of the uncertainty of their infectious status, pediatric patients may therefore be a potential source of infection in the dental practice and endanger health of the dental team and other patients.^13,14^

Due to the past critical situation of the COVID-19 pandemic, new guidelines for infection control in dental offices were published by the WHO and the Center for Disease Control and Prevention (CDC).^15,16^ The new recommendations include, for instance, the use of hand-sanitizing solutions for patients and caregivers, personal protective equipment (PPE) such as filtering facepiece (FFP) masks, goggles/face shields, protective clothing for professionals, and preoperative rinsing with mouthwash for patients. Old procedures such as isolation of the operative field using rubber dams, the use of high-pressure suction systems and non-rotary instruments for caries removal were also recommended to avoid the spread of pathogens and reduce the risk of cross-infections.^15-17^ The need to mitigate the aerosol spread and airborne transmission of SARS-CoV-2 in dental offices has led to more conservative dental procedures. In this attempt, the minimal intervention dentistry (MID), including atraumatic restorative treatments (ARTs) with manual caries removal, topical application of fluoride, and the use of silver diamine fluoride (SDF) has been recommended for caries management.^18-21^

Although COVID-19 has laid a significant burden on dental professionals, to date, the literature has a limited number of studies evaluating its impact on pediatric dentists,^22-26^ and to the best of our knowledge, no cross-country studies are available on this topic. In light of the ongoing global COVID-19 concern, this study sought to elucidate the impact of the pandemic on the management of biological risks and clinical strategies within pediatric dentistry through an online anonymous questionnaire. Therefore, the primary aim was to assess and compare the adherence of pediatric dentists in Brazil and Italy to the biosafety guidelines and operative protocols recommended by health authorities during the COVID-19 pandemic. Additionally, as a secondary aim, the study categorized participants based on their exposure to infection risks, thus delineating their distinct profiles.

## Methods

### Study design and ethics approval

This cross-country observational study was approved by the Ethics and Institutional Review Boards of the two countries (CAAE: 34299620.4.0000.5418; IRB-BURLO 10/2020), and the protocol followed the STROBE guidelines.

The study was conducted from July 14^th^ to August 17^th^, 2020 in Brazil (first wave of the COVID-19 pandemic) and from February 14^th^ to August 27^th^, 2021 in Italy (second wave of the pandemic) in full accordance with the principles expressed by the Helsinki Declaration.

### Participants

Eligible subjects were selected according to the following criteria. The target total sample size was based on 8033 pediatric dentists in Brazil and 5476 in Italy, considering a margin of error of 5% in a 95% confidence interval. Minimal samples of 367 and 236 participants, respectively, were estimated.

Inclusion criteria were pediatric dentists, aged 20-60 years, both genders, working in the National Health Service (NHS) and/or in private practice, residents and regularly registered with national dental councils in Brazil or Italy.

Exclusion criteria were incomplete forms and refusal of consent to participate in the study and data management in accordance with data protection laws by eligible participants. The consent form was made available online before the questionnaire; if individuals did not provide their consent, the questionnaire was immediately blocked and it was no longer possible to fill it out. Pediatric dentists residing or practicing dentistry outside the geographic bounds of Brazil and Italy were also excluded.

### Data collection

An online anonymous questionnaire was made available to all the eligible Brazilian and Italian pediatric dentists. To develop the survey, authors referred to the COVID-19 recommendations expressed by health authorities.^15,16^ The questionnaire, consisting of 62 questions, was administered in both Portuguese and Italian languages. The translation and adaptation were conducted by Italian researchers who received the English version of the original form. The questionnaire was designed in the Google Forms platform by combining open-ended, closed-ended, and multiple-choice questions following the Checklist for Reporting Results of Internet E-Surveys (CHERRIES).^27^ The 62 questions were organized in the following 7 domains: a) demographic and occupational data; b) organization of the dental practice; c) biological risk management protocols; d) behavior handling of pediatric patients; e) management of dental appointments; f) clinical operative protocols for pediatric patients; and g) means of communications with patients/guardians.

Data described in this manuscript referred to 34 of the 62 total survey questions from the following domains: a) demographic and occupational data; b) organization of the dental practice; c) biological risk management protocols, and 6) clinical operative protocols during the COVID-19 pandemic.

Before starting this study, a pilot study was carried out in a sample of 20 volunteers in each country^28^ to validate the content and reliability of the questionnaire. Participants of the pre-test were not included in the study sample. Professionals were contacted via email by dental councils and social media (WhatsApp, Facebook, and Instagram) using a snowball convenience sampling strategy.

### Statistical analysis

Exploratory statistical analysis was performed using the SPSS 28.0 software by an applied statistics specialist (PMC) and consisted of means and percentages; an alpha level of 0.05 was adopted. The association between categorical variables was tested using the Chi-square test.

A multivariate analysis (cluster analysis) was employed to categorize individuals into either a safer or the less safe group concerning the risk of COVID-19 they were subjected to based on the many variables collected from the participants. For that, groups (clusters) were generated with similar individuals within the same cluster and dissimilar to the individuals of the other cluster based on how closely associated they were. A two-step method was applied to assess profiles of health professionals according to sociodemographic aspects and the adopted biosafety protocols.^29^ The analysis included the following categorical variables: gender, business organization (public/private/both), dental education level (postgraduate level), years of working experience, use of disposable gown/jumpsuit, shoe and hair covers, sanitizing mat, eye protection (goggles), face shield, high-pressure suction, preoperative mouthwash, MID approaches for dental caries management, and type of mask, e.g. cloth, surgical, N95, or PFF. The final number of clusters was based on the interpretability and reliability of the cluster solution, by assessing the fit using the Bayesian Information Criterion (BIC) and quality of the analysis (silhouette coefficient).

## Results

A total of 643 participants completed the survey, but 2 of them were excluded for being foreigners (Mexican and Iranian); thus, the final sample consisted of 641 volunteers (377 Brazilians and 264 Italians) with no missing data.

Table 1 shows the sociodemographic and professional profile of the participants. Among Brazilians, the majority of professionals were females (94.0%), predominantly aged between 20 and 29 years (30.0%), and had more than 10 years of professional experience (58.0%). Within the Italian participants, 70.0% were females, mostly aged 30 to 39 years (32.9%), and 48.5% reported having over a decade of professional experience. Of participants who reported contracting SARS-CoV-2 in their workplace, 5.0% were Brazilian and 12.5% were Italian.

**Table 1:** Sociodemographic characteristics and professional background of participants in Brazil (n = 377) and in Italy (n = 264). Data are expressed as numbers and [percentages].

Variables	Brazil		Italy

	n [%]		n [%]
Northeast	61 [16]	Northeast	135 [51.1]
North	12 [3]	Northwest	55 [20.8]
Central-West	28 [8]	Center	35 [13.3]
South	54 [14]	South	15 [5.7]
Southeast	222 [59]	Islands	24 [9.1]
20-29	112 [30]		42 [15.9]
30-39	106 [28]		87 [32.9]
40-49	92 [24]		44 [16.7]
50-59	52 [14]		65 [24.6]
≥60	15 [4]		26 [9.9]
Female	353 [94]		184 [69.7]
Married/ cohabiting couple	223 [59]		127 [48.1]
Single	149 [40]		99 [37.5]
Widower	5 [1]		1 [0.4]
Others	-		37 [14]
Monthly household income (minimum wages )			
< R$ 6060	154 [41]	< €1500	23 [9]
R$ 6061 – R$ 12.120	116 [31]	€1.501 – 5.499	154 [58]
> R$ 12.120	107 [28]	> €5.500	26 [10]
-	-	Did not inform	61 [23]
Years of experience			
0 – 5	114 [30]		97 [36.7]
6 – 10	46 [12]		39 [14.8]
Over 10	217 [58]		128 [48.5]
Dental educational level			
Residency program	3 [1]	Residency program	-
*Lato Sensu*	194 [51]	*Lato Sensu*/ PhD	106 [40.2]
Master degree	66 [18]	Master degree	51 [19.3]
PhD	75 [20]	-	-
Bachelor	31 [8]	Bachelor	107 [40.5]
Not informed	8 [2]	Not informed	-
Business organization			
Public facilities	38 [10]		23 [8.7]
Private facilities	254 [67]		180 [68.2]
Both	85 [23]		61 [23.1]
Exposure to patients suspected of being infected with coronavirus			
Yes	102 [27]		120 [45]
Confirmed COVID-19 infection			
Yes	20 [5]		33 [12.5]
Dental hygienist in the staff			
Yes	237 [63]		192 [72.7]

In the combined cohort of participants from both countries, no statistically significant correlation was observed between SARS-CoV-2 infection and variables such as gender, level of dental education, organizational sector (public or private), years of professional experience, designation as a frontline dentist, or willingness to adopt biosafety measures after the pandemic (p > 0.05). Over 95% of professionals from both countries affirmed their capacity to effectively communicate pertinent information concerning COVID-19 infection and preventive measures with their patients or their parents.

The biosafety protocols implemented in pediatric dental offices are shown in Table 2. Brazilian professionals with extensive experience showed a higher propensity to adopt biosafety measures even after the pandemic. Moreover, professionals with over 10 years of experience employed three or more adjustments in their dental offices (Brazilians = 51.5%; Italians = 47.0%).

**Table 2:** Description of biosafety measures adopted by pediatric dentists in Brazil and Italy during the COVID-19 pandemic. Data are shown as percentages.

Variables	Brazil					Italy		
Dental office facilities								
Years of experience		**0–5**	**6–10**	**> 10**		**0–5**	**6–10**	**> 10**
Adjustments in dental office								
None	1.1	0.5	0.3	0.3	0.8	0.8	-	-
1–2 adjustments	18	11.1	1.1	5.8	3	1.1	0.4	1.5
3 or more adjustments	80.9	18.6	19.3	51.5	96.2	34.8	14.4	47
Hand-sanitizing solution for patients/caregivers								
Yes	93.1	27.1	11.1	54.9	99.2	35.9	14.8	48.5
Sanitizing mat								
Yes	48	12.2	4.5	31.3	48.5	16.7	6.8	25
Management of biological risk protocols								
Type of mask								
Surgical mask	9.3	3.2	0.8	5.3	2.6	1.1	0.4	1.1
N95-equivalent mask (PFF 1, 2 or 3)	19	5	1.3	12.7	23.9	5.7	3.4	14.8
N95 mask	23.3	7.7	1.3	14.3	7.2	2.3	1.1	3.8
N95-equivalent mask or N95 + surgical mask	47.8	14.1	8.8	24.9	66.3	27.7	9.8	28.8
Cloth	0.6	0.3	0	0.3	-	-	-	-
Clothing								
None	0.5	0.5	0	0	0.4	0.4	-	-
Disposable gown	18.5	5.8	0.5	12.2	31.8	12.5	5.3	14
Reusable fabric gown	8.5	3.7	0.8	4	37.9	15.1	4.2	18.6
Disposable gown + reusable fabric gown	65	19.1	10.1	35.8	15.9	5.7	2.3	7.9
Jumpsuit	4.8	0.5	0.5	3.8	14	3	3	8
Impermeable gown	2.7	0.5	0.3	1.9	-	-	-	-
Discard of disposable gowns								
After each patient	54.4	12.5	6.1	35.8	36	10.6	5.3	20.1
At the end of the day	28.9	11.1	4	13.8	33.7	14	4.9	14.8
When dirty	5.6	2.1	0.8	2.7	5.3	1.5	0.8	3
Does not wear a disposable gown	11.1	4.5	1.3	5.3	25	10.6	3.8	10.6
Discard of jumpsuit								
After each patient	3.4	0	0.5	2.9	8.3	0.8	2.3	5.3
At the end of the day	6.4	2.1	0.8	3.5	14.5	3.8	2.7	8
When dirty	0.8	0	0	0.8	1.5	-	-	1.5
Does not wear jumpsuit	89.4	28.1	10.9	50.4	75.7	32.2	9.8	33.7
Disposable shoe covers								
Yes	58.1	16.4	7.2	34.5	24.6	6.4	3.4	14.8
Hair cover								
Disposable hair cover	89.4	28.4	10.6	50.4	26.1	8.3	3.8	14
Fabric hair cover	9.8	1.6	1.6	6.6	57.6	22.4	9.8	25.4
Jumpsuit hood	-	-	-	-	10.2	2.3	1.1	6.8
None	0.8	0.3	0	0.5	6.1	3.8	-	2.3
Discard of hair cover								
After each patient	34	5.6	4	24.4	28	7.2	4.5	16.3
At the end of the day	55.7	23	6.4	26.3	33.7	11.7	4.6	17.4
Does not wear disposable hair cover	10.3	1.6	1.8	6.9	38.3	17.8	5.7	14.8
Discard of fabric hair cover								
After each patient	5.3	1	0.8	3.5	3	0.8	1.1	1.1
At the end of the day	15.1	3.7	1.6	9.8	66.7	25.4	10.2	31.1
Does not wear fabric hair cover	79.6	25.5	9.8	44.3	30.3	10.6	3.4	16.3
Eye protection (goggles)								
Yes	86.7	26	11.1	49.6	72.4	27.3	9.5	35.6
Face shield								
Yes	95.3	28.4	11.7	55.2	93.2	34.5	14.4	44.3
Dental hygienist in the staff								
Yes	62.9	15.4	8.5	39	72.7	28.8	12.1	31.8
High-pressure suction								
Yes	75.3	22.8	9.3	43.2	99.6	36.7	14.4	48.5
Disinfection of dental handpieces after use								
Disinfection	60.7	23.3	8.8	28.6	25	9.5	3.8	11.7
Sterilization	39.3	6.9	3.4	29	75	27.3	11	36.7
Preoperative mouthwash								
Yes (chlorhexidine/peroxide/povidone)	57	15.4	4.5	37.1	90.5	34	12.1	44.4
Willing to adopt biosafety recommendations after pandemic								
Yes	85.7	83	84.8	92.2	85.2	28	14	43.2

Regarding caries management, almost all Brazilian (82.9%) and Italian (95.4%) professionals reported being aware of the importance of performing MID to control cross-contagions during dental treatments and 92.0% and 80.7%, respectively, declared that they had been using these strategies daily. In Brazil, the predominant MID approaches employed included selective caries removal using non-rotary instruments, followed by atraumatic restorative treatment (ART), fluoride application (varnish/gel), and application of dental sealants. In Italy, the most frequent approaches were: application of dental sealants > fluoride application > selective caries removal using non-rotatory instruments > ART, and finally, ozone therapy. The MID approaches used in both countries are illustrated in Figure.

**Figure:**
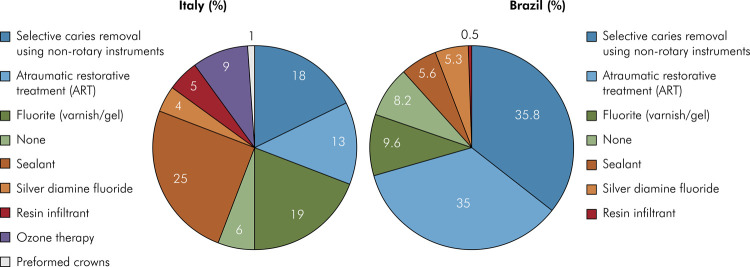
Minimal intervention dentistry approaches performed by dentists during the COVID-19 pandemic in Italy and Brazil. Data are expressed as percentages of the total answers collected.

Table 3 shows the taxonomy of the two clusters generated by the two-step cluster analysis, which describes the two profiles of participants according to their adherence to the recommended biosafety measures: the “safe” group (n = 219) and the “less safe” group (n = 422). The “safe” group was characterized by a greater number of professionals who adopted biosafety measures during the COVID-19 pandemic, such as use of N95 masks, discarding of the disposable gown/jumpsuit/hair/shoe cover after each patient, pre-operative chlorhexidine/peroxide/povidone mouthwash, and MID approaches. Moreover, the safe group was composed mainly of professionals with more years of experience employed in public facilities. The safe group had a lower percentage of professionals with postgraduate degrees (54.3%) than the less safe group (71.8%); 38.7% and the 27.7% of the Brazilian and Italian participants, respectively, were included in this cluster.

**Table 3:** Taxonomy description of clusters and centroids. Data are expressed as percentages.

Variables	Cluster 1	Cluster 2
Proposed taxonomy	Less safe group	Safer group
Number of participants	422	219
Gender		
Females	83	85
Years of experience		
< 0	6.6	2.7
1–5	34.3	14.6
6–10	13.5	12.8
> 10	45.5	69.9
Business organization		
Private facility	75.4	53.4
Public facility	5.9	16
Both	18.7	30.6
Post-graduate degrees	71.8	54.3
Discard of disposable gown/ jumpsuit after each patient	25.6	91.8
Eye protection (goggles)	79.6	83.1
Face shield	92.7	97.7
Minimal intervention dentistry approaches	82.9	95.4
Sanitizing mat at the entrance of dental office	42.9	58.5
Pre-operative chlorhexidine/peroxide/povidone mouthwash	63.5	76.3
Type of mask		
Cloth	0.5	0
Surgical	8.3	3.2
N95 or equivalent (PFF)	91.2	96.8
Disposable shoe covers	34.1	63.9
Discard of disposable hair cover after each patient	1.4	89.5
Brazil	61.3	38.7
Italy	72.3	27.7

Two-step cluster; BIC = 9009.765; Silhouette coefficient analysis: moderate quality.

## Discussion

The sudden appearance of the COVID-19 pandemic has greatly changed dental practices and attitudes worldwide, including in the pediatric field. The critical situation has demanded the adoption of extra care and measures in dental offices as well as minimal or noninvasive operative techniques to contain the dissemination of the virus.^15-17^ Given the current situation, the present study aimed to deepen the knowledge on the pandemic’s impact on biological risk management and clinical approaches in the pediatric dental field among Brazilian and Italian dentists through an online anonymous questionnaire. To the best of our knowledge, this is the first cross-country survey on this topic and could be therefore considered a guiding study in the field.

Of the 641 included dentists (Brazilian sample = 377 participants and Italian sample = 264 participants), the female gender was the most represented in both samples: 69.7% and 94.0%, respectively, Brazilian and Italian participants were females, highlighting the predominance of female dentists in pediatric dentistry. A greater number of young subjects was also found for both samples (age group 20-29 years = 112 Brazilian participants; age group 30–39 years = 106 Italian participants) probably due to the online diffusion of the questionnaire.

Southern regions of Brazil and northern regions of Italy had higher response rates. This geographical bias could be linked to the location of the two primary health-promoting centers, which are situated in the South of Brazil and the North of Italy. Moreover, these disparities in response rates may suggest an unequal distribution of dentists, especially across Brazil’s national territory.^30,31^ The Southeast region of Brazil, with approximately 86.3 million inhabitants,^32^ accounted for the largest proportion of respondents in this study. Additionally, observations from Italian respondents underscored the different distribution of dentists across Italy’s regions, which is typically concentrated in urban areas and regions with larger populations due to higher population density and increased demand for healthcare services. In contrast to other occupational data, the majority of participants in both samples had over ten years of professional experience (Brazilian participants = 58.0%, Italian participants = 48.5%) and reported working in the private sector (Brazilian participants = 67.0%, Italian participants = 68.2%). Notably, these findings align with the distribution of dentists across Italy’s territory, where the concentration of experienced professionals and those employed in the private sector closely matches the surveyed population.

Regarding organization of dental practices and adherence to biosafety measures recommended by global health authorities, both samples reported significant adjustments within their dental offices and an intensified implementation of disinfection protocols in accordance with health authority guidelines.^15-16^ These adjustments were notably consequential, given the low rates of contagion among participants, with 5.0% of Brazilian dentists and 12.5% of Italian dentists affected. These data underscore the efficacy of the recommended measures and protocols. From these findings, two meaningful interpretations emerge: the contamination of pediatric dentists by SARS-CoV-2 might have occurred during the doffing of the PPE or the exposure might have taken place outside the workplace.

In terms of biosafety measures embraced by dentists in relation to their professional experience, an interesting trend emerged: professionals with extensive experience reported a higher frequency of adjustments within their dental offices, with a greater propensity to adopt recommended protocols, highlighted by the cluster analysis. Particularly, disparities were observed in the choice of PPE between Brazilian and Italian professionals, revealing financial inequities particularly among Brazilian dentists. This divergence was reinforced by the fact that the majority of Brazilian participants reported lower monthly income compared to their Italian counterparts, reflecting the different economies of Brazil, categorized as an upper middle-income economy, and Italy, a high-income economy.^33^

The prevailing intention among the majority of dentists in both countries (85.7% Brazilians and 85.2% Italians) to maintain these implemented biosecurity measures even after the pandemic emphasizes the steadfast commitment of pediatric dentists in preventing viral transmission until herd immunity is achieved. This positive finding highlights the dedication of pediatric dentists to long-term preventive measures in combating the spread of the virus.

In the domain of MID, a significant majority of professionals reported the adoption of minimally invasive operative protocols to preserve tooth tissues while mitigating viral spread, which is imperative in the COVID-19 era.^18-21^ The preferred techniques varied among Brazilian and Italian dentists, with selective caries removal using non-rotatory instruments (35.8%), ART (35.0%), and application of fluoride as varnish/gel (9.6%) predominating in Brazil versus fissure sealants (25.0%), application of fluoride (19.0%), and ART (13.0%) in Italy. Among the less used techniques in both countries was the use of silver diamine fluoride (SDF) for arresting active caries lesions probably due to the non-esthetic black discoloration caused by the silver phosphate precipitation in the treated tissues.^20,21^ Ozone therapy was an alternative minimally invasive option for dental caries treatment in Italy but not in Brazil.^34^

The study had limitations and strengths. Limitations included low response rates from professionals, which resulted in moderate sample sizes and the fact that, as for all cross-sectional surveys, data were collected in a concise period. Another notable limitation may be the fact that, despite recommendations of the world health authorities,^15,16^ participants could have faced different restrictions in providing pediatric dental care depending on local and statewide restrictions and the official advice to postpone non-emergency dental care.^35-37^ In addition, it has to be considered that some parents might have hesitated to take their children to healthcare services because they believed there could be a risk of infection.^38,39^

The use of constructed surveys is recognized as a rapid and valuable method to efficiently gather data, especially when addressing large-scale research endeavors.^40^ The dissemination of the questionnaire via email and social networks allowed authors to promptly access a sample that was as representative as possible of pediatric dentists in Brazil and Italy, recognized as pivotal epicenters of the pandemic in Latin America and Europe, respectively. The nationwide scope of this study, coupled with data collection during distinct pandemic waves in Italy and Brazil, stands as a significant strength. These factors allowed a comprehensive understanding of the COVID-19 impact on biological risk management and clinical practices within pediatric dentistry in the studied countries. Additionally, Italy and Brazil, as epicenters of the pandemic in Europe and South America, provided a unique cross-country perspective, further emphasizing the study’s uniqueness.

According to the obtained findings, participants of the two countries were following the main recommended biosafety measures, with no socio-demographic or cross-cultural differences.

Given the significance of the pandemic, further studies with a larger sample size and a longer follow-up period are needed to gain more detailed findings. Knowing and comparing the adherence to COVID-19 guidelines of the world health authorities in different countries will allow researchers and professionals to get a picture of the impact of this pandemic worldwide. Moreover, conducting another survey after the pandemic would be insightful to assess and compare the shifts in the landscape between the pandemic and the current scenario.

## Conclusions

Two different profiles of pediatric dentists were identified based on the biosafety protocols adopted during the COVID-19 pandemic, the “safer” and the “less safe” groups among Brazilian and Italian participants. In addition, changes were implemented in the dental care of children, with a focus on adopting minimal intervention dentistry.
